# Outbreak of infections by *Mycobacterium abscessus* subsp. *abscessus* associated with cosmetic surgeries in the Brazil/Paraguay border: a binational cohort study, 2021-2022

**DOI:** 10.1590/S2237-96222025v34e20250516.en

**Published:** 2025-09-08

**Authors:** Mateus de Paula von Glehn, Griselda Salcedo, Talita Gomes da Silva Batista, Lorena Elizabeth Romero Caballero, Carlos Henrique Michiles Frank, Eunice Atsuko Totumi Cunha, Aline Schio de Souza, Camile de Moraes, Igor Gonçalves Ribeiro

**Affiliations:** 1 Secretaria de Estado da Saúde do Distrito Federal, Brasília, DF, Brazil; 2 Ministerio de Salud Publica y Bienestar Social, Field Epidemiology Training Program, Asunción, Paraguai; 3 Ministério da Saúde, Programa de Treinamento em Epidemiologia Aplicada aos Serviços do SUS, Brasília, DF, Brazil; 4 Laboratório Central de Saúde Pública, Campo Grande, MS, Brazil; 5 Secretaria de Estado de Saúde do Mato Grosso do Sul, Campo Grande, MS, Brazil; 6 Fundação Oswaldo Cruz, Núcleo de Epidemiologia e Vigilância em Saúde, Brasília, DF, Brazil

**Keywords:** Nontuberculous mycobacteria, Field Epidemiology, Surgical Wound Infection, Medical Tourism, Cohort Studies, Micobacterias no Tuberculosas, Epidemiología de Campo, Infección de Heridas Quirúrgicas, Turismo Médico, Estudios de Cohorte

## Abstract

Report on a field investigation into the outbreak of skin infections by *Mycobacterium abscessus* subsp. *abscessus* following cosmetic procedures.

A retrospective cohort study was conducted using secondary data from the Epidemiology Program Applied to SUS Services (EpiSUS) about the investigation conducted in 2022, in partnership with Paraguay’s Field Epidemiology Training Program. Clinical, laboratory, and environmental information and surgical records were analyzed to characterize the cases, define the exposed population, and analyze the risk. Relative risk (RR) was estimated with a 95% confidence interval (95%CI) and Fisher’s exact test was used for statistical evaluation.

Of the 108 individuals who underwent surgery in the period investigated, 10 became ill. Relative risk of illness was 6.50 (95%CI 2.28; 18.49; p-value 0.003) for surgeries performed in the critical period (December to January) and 10.29 (95%CI 1.37; 77.19; p-value 0.004) for lipoabdominoplasty.

*Mycobacterium abscessus* subsp. *abscessus* outbreak occurred among women undergoing cosmetic surgeries performed by the same professional in a hospital in Paraguay, with a higher risk associated with a specific period and type of procedure. Our findings highlight the importance of health surveillance in bordering regions and international cooperation in response to cross-border events. Educational, regulatory, and inspection strategies targeting medical tourism are essential to prevent new events.

## Resumen

Reportar una investigación de campo del brote de infecciones cutáneas por *Mycobacterium abscessussubsp.abscessus* después de procedimientos estéticos.

Se trata de un estudio de cohorte retrospectivo con datos secundarios del Programa de Epidemiología Aplicada a los Servicios del SUS (EpiSUS), referido a la investigación realizada en 2022 en colaboración con el *Field Epidemiology Training Program* del Paraguay. Se analizaron los datos clínicos, de laboratorio, ambientales y los registros quirúrgicos para caracterizar los casos, definir la población expuesta y análisis de riesgo. El riesgo relativo (RR) se estimó con un intervalo de confianza del 95% (IC 95%), y se utilizó la prueba exacta de Fisher para la evaluación estadística.

108 personas se sometieron a cirugía en el período investigado, de las cuales 10 enfermaron. El riesgo relativo de enfermedad fue de 6,50 (IC 95% 2,28; 18,49; p-valor 0,003) entre las cirugías realizadas en el período crítico (diciembre a enero) y de 10,29 (IC 95% 1,37; 77,19; p-valor 0,004) para la lipoabdominoplastia.

El brote ocurrió entre mujeres sometidas a cirugías estéticas realizadas por el mismo profesional en un hospital de Paraguay, con un mayor riesgo asociado a un período específico y un tipo de procedimiento. Los hallazgos destacan la importancia de la vigilancia sanitaria en las zonas fronterizas y de la cooperación internacional para responder a los eventos transfronterizos. Las estrategias educativas, regulatorias y de inspección dirigidas al turismo médico son fundamentales para prevenir nuevos eventos.

## Ethical aspects

**This research respected the ethical principles, having obtained the following approval data:**

Research Ethics Committee

Comissão Nacional de Ética em Pesquisa

Opinion number

5.928.114

Approval date

7/3/2023

Certificate of Submission for Ethical Appraisal

66265222.8.0000.0008

Informed Consent Form

Use of anonymized secondary data hindered obtaining informed consent. The activities took place during a public health emergency, focusing on responding to the event and observing the principles of privacy and confidentiality, according to Resolution No. 674/2022.

## Introduction

Nontuberculous mycobacteria are acid-fast bacilli (AFB) widely found in the environment. Capable of forming biofilms on organic and inorganic surfaces that are difficult to remove, they are resistant to various chemical disinfection methods (1-3). Nontuberculous mycobacteria can be classified according to their growth time in culture media, divided into slow-growing mycobacteria (more than 7 days) and fast-growing mycobacteria (less than 7 days) (4,5).

Infections by fast-growing mycobacteria have been reported in Brazil associated with surgical procedures, especially those employing video equipment (4,6). Among the fast-growing mycobacteria described are the species belonging to the *M. abscessus* complex: *M. abscessus* subsp. *abscessus*, *M. abscessus* subsp. *massiliense* and *M. abscessus* subsp. *bolettii*. Outbreaks reported in Brazil describe infections by *M. massiliense* which so far seems to be the predominant subspecies. Of the three known subspecies, *M. abscessus* has the highest antimicrobial resistance profile (7).

On 7 April 2022, the Health Surveillance of Mato Grosso do Sul issued a technical alert about an outbreak of *M. abscessus* infections in people undergoing cosmetic procedures in Pedro Juan Caballero, Paraguay. The situation prompted a joint investigation by the field epidemiology training programs of Brazil and Paraguay. All cases were linked to a single hospital and surgeon.

This study reports on a field investigation into an outbreak of skin infections by *Mycobacterium abscessus* subsp. *abscessus* following cosmetic procedures.

## Methods

### Design

A retrospective cohort study was conducted using secondary data from the Epidemiology Program Applied to SUS Services (EpiSUS) about the investigation conducted in 2022, in partnership with Paraguay’s Field Epidemiology Training Program. The data contained in the database was obtained during a field epidemiological investigation conducted as part of a public health surveillance campaign.

### Context

In April 2022, Brazil’s National Focal Point for the International Health Regulations identified a rumor about post-surgical infections in patients undergoing plastic surgery at a hospital in Pedro Juan Caballero, Paraguay. Confirmation by the Health Surveillance of Mato Grosso do Sul and the National Focal Point for Paraguay’s International Health Regulations prompted a joint investigation. The EpiSUS team was invited by the Paraguayan team to participate in the field activities. The investigation took place in Pedro Juan Caballero, department of Amambay, a city on the dry border with Ponta Porã, in the state of Mato Grosso do Sul, a region of intense cross-border flow and epidemiological relevance.

Biological samples obtained from the skin lesions were sent to the Central Public Health Laboratory of Mato Grosso do Sul (LACEN), where they were processed by cultivation in solid medium (Lowenstein-Jensen) and liquid medium (MGIT broth). Molecular identification was performed by line probe assay (LPA) hybridization. Subsequently, the samples were sent to the reference laboratory of the Oswaldo Cruz Foundation (FIOCRUZ) for confirmation and clonality assessment. Water samples collected at the establishment were also processed at LACEN, with microbiological analysis and an attempt to isolate fast-growing mycobacteria.

### Participants

Individuals who underwent plastic surgery from August 1^st^, 2021 and April 30^th^, 2022 in a private hospital in Pedro Juan Caballero.

### Variables, data sources, and measurement

The data source used in this study was an anonymized database made available by EpiSUS. Data were collected during a field epidemiological investigation conducted during a public health emergency, as part of regular health surveillance actions.

Throughout the investigation, the study population was defined from a list of 108 people who underwent cosmetic surgery from August 2021 to April 2022, provided by the professional responsible for the procedures. The list included name, type of surgery, date of procedure, and contact number.

Based on this information, the teams drew up a bilingual questionnaire for interviews, which covered: identification (name, date of birth, address); sociodemographic data (sex, ethnicity/skin color, occupation, schooling level, income); health history (comorbidities, medication use); surgery data (date, time, duration, type); clinical picture (date of symptom onset, signs and symptoms presented), and laboratory information (date of collection, type of sample, laboratory method and microorganism isolated).

Individuals were contacted by telephone and invited to take part in the interview, which was conducted jointly by researchers from Paraguay and EpiSUS. Paraguayan citizens were contacted by the Paraguayan team and Brazilian citizens by EpiSUS. Several contact attempts were made on different days and at different times, with no pre-established limit. As a sensitization strategy, a contact was sent in advance via messaging app introducing the research team, the objectives of the study and scheduling the interview.

Each investigator made at least three attempts to contact each individual, either by phone or app. Unsuccessful contact attempts were considered losses. In addition to the interviews, secondary data from the health inspection conducted by the Paraguayan health authority was used.

The cohort study included 75 people (10 confirmed cases and 65 asymptomatic). Possible associations between illness and socioeconomic/demographic and exposure variables were tested. The database used in the study can be accessed in the SciELO Data repository (https://data.scielo.org/).

### Statistical analysis

Numerical variables were described using measures of central tendency and dispersion. Categorical variables were presented as absolute frequencies. Relative risk (RR) was the measure of association used, and the associations were assessed for statistical significance.

Associations between categorical variables were analyzed using Fisher’s exact test. Statistical analyses were performed using Epi Info software version 7.2.5.0, and the graphs were drawn up using Microsoft Excel 2016.

## Results

**Figure 1.**
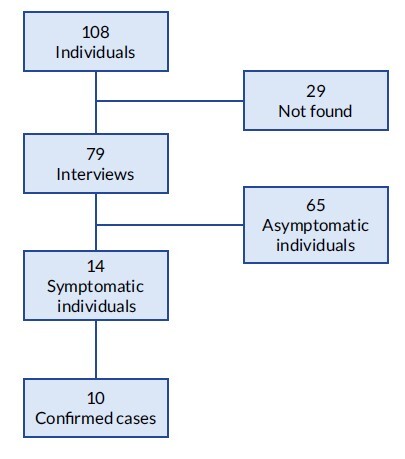
Study population, interviews conducted and clinical status according to symptoms. Paraguay, 2021-2022 (n=108)

From August 2021 to April 2022, 108 surgeries were performed by the same surgeon in a private hospital located in Pedro Juan Caballero city. A total of 14 individuals (12.9%) had symptoms compatible with infection by fast-growing mycrobacteria (abscess, fistula, suture dehiscence). Of these, 10 (9.3%) had *Mycobacterium abscessus* subsp. *abscessus* infection confirmed by laboratory tests and all were interviewed. The remaining four symptomatic individuals did not undergo sample collection for laboratory diagnosis. Of the 94 asymptomatic clients, 65 (69.1%) were located and interviewed (Figure 1).

**Figure 2.**
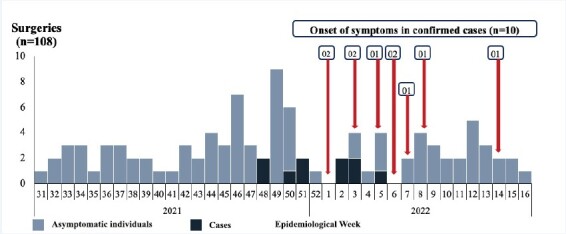
Distribution of cosmetic surgeries, confirmed cases and symptoms onset according to epidemiological week. Paraguay, 2021-2022 (n=108)

In December 2021, 21 surgeries were performed (19.4%), with four confirmed cases (0.19 incidence). In January 2022, 11 surgeries were performed (10.2%), of which five evolved to confirmed cases (0.45 incidence) (Figure 2).

All confirmed cases (n=10) occurred in women aged 30 to 46 years (Median=34; Q1=33; Q3=42), with no reported comorbidities. Nine were Brazilian and one Paraguayan. Most had tertiary education (n=5) or graduate studies (n=1), and the reported income ranged from up to 2 minimum wages to more than 5 to 10 minimum wages (four did not inform). As for ethnicity/skin color, eight self-declared themselves white and two were mixed-race (Table 1).

Symptom onset occurred from 3 to 72 days after surgery, with a median of 31 days (Q1=21; Q3=65). The onset of the first case was on January 5, 2022, 36 days after lipoabdominoplasty and placement of breast prostheses. The last case was identified on April 6, 2022, with a history of lipoabdominoplasty performed on January 31 (65 days of incubation).

Clinical presentations included formation of hyperemic subcutaneous nodules evolving to violet coloration, fistulas, and purulent drainage, compatible with granulomatous reaction. Of the 10 patients, eight made empirical use of antimicrobials prior to laboratory confirmation. Table 2 describes the antimicrobials used, clinical signs and symptoms, patient age and the incubation period of the cases.

Sensitivity profile of the isolated strains identified resistance to clarithromycin, doxycycline, sulfamethoxazole-trimethoprim, linezolid, ciprofloxacin and moxifloxacin, and intermediate resistance to imipenem and cefoxitin. Strains were sensitive to amikacin, tigecycline and bedaquiline.

Based on the antibiogram, treatment was prescribed with bedaquiline (100 mg: 4 tablets/day for 14 days, followed by 2 tablets/day three times per week for 24 weeks, orally); amikacin (500 mg: 2 ampoules/day for 14 days, followed by 2 ampoules/day three times per week for 8 weeks, intravenously), and tigecycline (50 mg: 2 ampoules on the first day, followed by 1 ampoule/day for 41 days, intravenously). Brazilian patients received treatment at the University Hospital of Dourados, Mato Grosso do Sul, whereas the Paraguayan patient was treated at the local hospital of Pedro Juan Caballero.

Relative risk of becoming ill was higher for surgeries performed in the critical period (December to January [RR 6.50; 95%CI 2.3; 18.5; p-value 0.003]) and for those who underwent lipoabdominoplasty (RR 10.29; 95%CI 1.4; 77.2; p-value 0.004) (Table 3).

FIOCRUZ performed clonality assessment by whole genome sequencing in samples obtained from the cases. Its report revealed a high level of genomic similarity between the isolates, with identification of the same Single Nucleotide Polymorphisms (SNP), deletions and insertions, indicating that the cases were related to the same clonal strain of *Mycobacterium abscessus* subsp. *abscessus.*

Results from environmental samples showed that the water used in the establishment was untreated and therefore not chlorinated. Total coliforms were found at two points (the reservoir outlet and one of the operating room sink taps) and *Pseudomonas aeruginosa* at another three points (the purge tap and two operating room sink taps). Aliquots of the sample were seeded for isolation of fast-growing mycobacteria, with negative results.

Inspection by the health authority of the Department of Amambay, Paraguay, pointed out non-conformities and suggestions for improvement. Among other information, the report produced by the Paraguayan inspection team indicates: weaknesses in hygiene protocols; incorrect handling of infectious waste; disorganization and lack of cleanliness in the sterilization room; lack of maintenance on air conditioning units; lack of an isolation room; existence of artificial flower arrangements; and inaccurate identification and labelling of sterilized materials.

On one of the visits, verbal report informed that the professional responsible for cleaning the materials left for health reasons in mid-December 2021, and was replaced by another worker.

## Discussion

This study describes an outbreak of *Mycobacterium abscessus* subsp. *abscessus* infections following cosmetic surgeries performed by a private hospital in Pedro Juan Caballero, Paraguay. Infection onset was associated with surgeries conducted from December 2021 to January 2022, and undergoing lipoabdominoplasty. Genomic analysis identified the same strain among the cases, reinforcing the hypothesis of a common source of infection.

Our results should be considered in light of the limitations inherent in using secondary data and a retrospective design, which may be subject to selection and memory biases. The definition of exposed population relied on records provided by the professional responsible for the surgeries, which may have led to underreporting of procedures. Information bias is also a possibility since data were collected by retrospective interviews, conducted in different contexts and with potential differences in understanding between the interviewees.

Association between the critical period (December–January) and the increase in cases suggests a one-off failure in the sterilization and infection control processes. Replacement of the professional responsible for cleaning the materials in December, reported during the health inspection, may have represented a moment of vulnerability. Proper cleaning of reusable materials, such as liposuction cannulas, requires technical knowledge and strict reprocessing protocols (8) which may not have been fully ensured in this context. Previous Brazilian studies have described outbreaks of fast-growing mycobacteria related to failures in the reprocessing of materials, highlighting the resistance of these mycobacteria to mechanical removal and to commonly used sanitizers such as glutaraldehyde (9-12).

The clinical pattern observed, with late onset symptoms and progression to fistulized lesions, is compatible with the behavior of infections caused by fast-growing mycobacteria after invasive procedures. The antimicrobial resistance found in the isolates reinforces the therapeutic challenges associated with these agents, as has already been described in outbreaks in Brazil from 1998 to 2017, in which treatment was prolonged and costly, often requiring reoperation (13). Restricted sensitivity to three antimicrobials—tigecycline, bedaquiline and amikacin—limited the therapeutic options available, requiring inter-institutional coordination to make treatment viable, especially in the case of Brazilian patients.

Genomic research indicated that the clinical isolates belonged to the same clonal strain, which corroborates the hypothesis of a common source. This finding is compatible with studies conducted in other settings, which have also identified clonality between clinical isolates in outbreaks related to cosmetic procedures (11,14,15). Although the environmental samples were negative for fast-growing mycobacteria, the identification of coliforms and *Pseudomonas aeruginosa* at critical points in the hospital facility suggests systemic failures in water sanitary control, which may have contributed to the contamination. The absence of chlorination in the hospital water reinforces this interpretation.

The growth of medical tourism, driven by factors such as lower costs, shorter waiting times and the search for discretion, has been accompanied by an increase in the number of adverse events (16,17). International reports of infection by fast-growing mycobacteria following plastic surgery in countries like the Dominican Republic and Mexico show similarities to this outbreak, including the demographic profile of the cases, type of procedure and the antimicrobial resistance of the isolates (18-20). Although Brazil has already registered cases of fast-growing mycobacteria infections after cosmetic procedures, no previous reports were found involving Brazilians exposed outside the national territory. The current outbreak is a wake-up call for the health authorities in both countries.

Elements in the outbreak investigated are characteristic of a healthcare-related infection, including an individual risk factor (type of surgery), a time factor (concentration of cases over a short period) and an environmental factor (inadequate infrastructure and sanitary practices). The cases may have presented polymicrobial infection initially, but the empirical use of antimicrobials before diagnostic confirmation may have contributed to the selection of *M. abscessus*, whose resistance to multiple therapeutic classes is documented (2,7).

In conclusion, this outbreak was associated with sanitary failures in a private health establishment, aggravated by the context of medical tourism. Our findings reinforce the need for effective regulatory mechanisms, surveillance and international co-operation to prevent adverse events in cross-border procedures.

**Table 1.** Sociodemographic characteristics of confirmed cases and asymptomatic individuals. Paraguay, 2021–2022 (n=75)

Parameter	Confirmed cases (n=10)	Asymptomatic individuals (n=65)	Total
	n	n	(n=75)
**Sex**			
Female	10	61	71
Male	-	4	4
**Age group (years)**			
Under 30	-	19	19
30 to 39	6	24	30
40 to 49	4	14	18
50 and over	-	7	7
Not informed	-	1	1
**Ethnicity/skin-color**			
White	8	37	45
Black	-	2	2
Asian	-	1	1
Mixed-race	2	22	24
Not informed	-	3	3
**Schooling level**			
Primary education	2	2	4
Secondary education	2	21	23
Tertiary education	5	37	42
Graduate studies	1	5	6
**Nationality**			
Brazilian	9	50	59
Paraguayan	1	15	16
**Income**			
Up to two salaries	3	11	14
More than 2 to 5	2	24	26
More than 5 to 10	1	5	6
More than 10	-	3	3
Not informed	4	22	26

**Table 2.** Confirmed cases according to age, incubation period, signs and symptoms presented and antimicrobial used. Paraguay, 2021-2022 (n=10)

Characteristic	Case									
	1	2	3	4	5	6	7	8	9	10
**Age (years)**	33	34	32	41	30	36	31	42	46	43
Incubation period(days)	36	21	30	3	20	70	36	72	65	21
**Signs and symptoms**	Painful nodule, hyperemia, increased local heat, fistula, secretion	Painful nodule, hyperemia, increased local heat, fistula, secretion	Painful nodule, hyperemia, increased local heat, fistula, secretion	Painful nodule, hyperemia, increased local heat, secretion, dehiscence	Painful nodule, hyperemia, local heat, fistula, secretion, dehiscence	Painful nodule, hyperemia, local heat, fistula, secretion, dehiscence	Local pain, hyperemia, local heat, fistula, secretion, dehiscence	Nodule (initially painless, evolving with local pain)	Fistula, secretion	Secretion, dehiscence
**Antibiotics used**	1. Clarithromycin 500mg 12/12h for 15 days 2. Cefuroxime 500mg 12/12h for 15 days 3. Ciprofloxacin 500mg 12/12h for 75 days 4. Clarithromycin 500mg 8/8h for 75 days 3. Fluconazole	1. Azithromycin 500mg 1x/day for 6 days	1. Ciprofloxacin 500mg 12/12h for 20 days 2. Clarithromycin 500mg 8/8h for 20 days 3. Fluconazole 150mg 1 capsule/week for 4 weeks	1. Amoxicillin/Clavulanic acid	1. Ciprofloxacin 500mg 12/12h for 30 days 2. Clarithromycin 500mg 8/8h for 30 days	1. Ciprofloxacin 500mg 12/12h for 20 days 2. Clarithromycin 500mg 12/12h for 20 days 3. Ciprofloxacin 500mg 12/12h for 30 days 4. Doxycycline 200mg 12/12h for 30 days	1. Sulfamethoxazole/Trimethoprim 12/12h for 15 days 2. Clarithromycin 500mg 8/8h for 15 days 3. Ciprofloxacin 500mg 12/12h for 15 days 4. Cefuroxime axetil 12/12h for 7 days 5. Metronidazole 400mg 8/8h for 7 days	1. Clarithromycin 500mg 8/8h for 14 days	No antibiotics use	No information available

**Table 3.** Association between sociodemographic characteristics, type of surgery, and occurrence of confirmed cases with relative risk (RR [95%CI]). Paraguay, 2021-2022 (n=75)

Characteristic	Confirmed cases	Asymptomatic individuals	RR (95%CI)	p-value
**Sex**				1.000
Female	10	61	0.90 (0.14; 5.87)	
Male	0	4		
**Income**				0.334
Up to two salaries	3	11	1.95 (0.76; 5.05)	
More than 2 minimum wages	3	32		
**Schooling level**				1.000
Primary/Secondary education	2	15	0.85 (0.19; 3.64)	
Tertiary education	8	50		
**Ethnicity/skin color**				0.302
White	8	37	2.40 (0.54; 10.48)	
Non-White	2	25		
**Critical period**				0.003
Yes	5	5	6.50 (2.28; 18.49)	
No	5	60		
**More than one procedure**				1.000
Yes	2	12	1.09 (0.26; 4.58)	
No	8	53		
**Type of surgery**				
**Lipoabdominoplasty**				0.004
Yes	9	26	10.29 (1.37; 77.19)	
No	1	39		
**Mastopexy**				1.000
Yes	1	8	0.81 (0.12; 5.69)	
No	9	57		
**Breast prosthesis**				0.480
Yes	2	23	0.50 (0.11; 2.18)	
No	8	42		

## Data Availability

The database used in the study can be accessed in the SciELO Data repository (https://data.scielo.org/).
