# Supplementary material for: Outbreak of infections by Mycobacterium abscessus subsp. abscessus associated with cosmetic surgeries in the Brazil/Paraguay border: a binational cohort study, 2021-2022
Source: Epidemiol Serv Saude. 2025 Sep 8;34:e20250516. doi: 10.1590/S2237-96222025v34e20250516.en (PMC12435886; doi:10.1590/S2237-96222025v34e20250516.en)
Supplement: Translation

# Translation: Surto de infecções por Mycobacterium abscessus subsp. abscessus associado à realização de cirurgias estéticas na região de fronteira Brasil/Paraguai: estudo de coorte binacional, 2021-2022

**DOI:** 10.1590/S2237-96222025v34e20250516.pt

**Authors:** Mateus de Paula von Glehn, Griselda Salcedo, Talita Gomes da Silva Batista, Lorena Elizabeth Romero Caballero, Carlos Henrique Michiles Frank, Eunice Atsuko Totumi Cunha, Aline Schio de Souza, Camile de Moraes, Igor Gonçalves Ribeiro

**Affiliations:** 1 Secretaria de Estado da Saúde do Distrito Federal, Brasília, DF, Brasil; 2 Ministerio de Salud Publica y Bienestar Social, Field Epidemiology Training Program, Asunción, Paraguai; 3 Ministério da Saúde, Programa de Treinamento em Epidemiologia Aplicada aos Serviços do SUS, Brasília, DF, Brasil; 4 Laboratório Central de Saúde Pública, Campo Grande, MS, Brasil; 5 Secretaria de Estado de Saúde do Mato Grosso do Sul, Campo Grande, MS, Brasil; 6 Fundação Oswaldo Cruz, Núcleo de Epidemiologia e Vigilância em Saúde, Brasília, DF, Brasil

**Keywords:** Micobactérias não Tuberculosas, Epidemiologia de Campo, Infecção da Ferida Cirúrgica, Turismo Médico, Estudos de Coortes

## Abstract

Relatar a investigação de campo de surto de infecções cutâneas por *Mycobacterium abscessus* subsp. *abscessus* após procedimentos estéticos.

Trata-se de estudo de coorte retrospectivo com dados secundários do Programa de Epidemiologia Aplicada aos Serviços do SUS (EpiSUS), referentes à investigação realizada em 2022, em parceria com o Field Epidemiology Training Program do Paraguai. Foram analisadas informações clínicas, laboratoriais, ambientais e registros cirúrgicos para caracterização dos casos, definição da população exposta e análise de risco. Estimou-se o risco relativo (RR) com intervalo de confiança de 95% (IC95%) e utilizou-se o teste exato de Fisher para avaliação estatística.

108 pessoas foram submetidas a cirurgias no período investigado, das quais 10 adoeceram. O risco relativo de adoecimento foi de 6,50 (IC95% 2,28; 18,49; p-valor 0,003) entre cirurgias realizadas no período crítico (dezembro a janeiro) e de 10,29 (IC95% 1,37; 77,19; p-valor 0,004) para lipoabdominoplastia.

O surto ocorreu entre mulheres submetidas a cirurgias estéticas realizadas por um mesmo profissional em hospital no Paraguai, com maior risco associado a um período específico e a um tipo de procedimento. Os achados destacam a importância da vigilância em saúde em áreas de fronteira e da cooperação internacional para resposta a eventos transfronteiriços. Estratégias educativas, regulatórias e de fiscalização voltadas ao turismo médico são fundamentais para prevenir novos eventos.

## Aspectos éticos

**Esta pesquisa respeitou os princípios éticos, obtendo os seguintes dados de aprovação:**

Comitê de ética em pesquisa

Comissão Nacional de Ética em Pesquisa

Número do parecer

5.928.114

Data de aprovação

7/3/2023

Certificado de apresentação de apreciação ética

66265222.8.0000.0008

Registro do consentimento livre e esclarecido

Foram utilizados dados secundários anonimizados, sem possibilidade de obtenção do TCLE. As atividades ocorreram em contexto de emergência em saúde pública, com foco na resposta ao evento, observando-se os princípios de privacidade e confidencialidade, conforme a Resolução nº 674/2022.

## Introdução

Micobactérias não tuberculosas são bacilos álcool-ácido resistentes (BAAR) amplamente presentes no ambiente. Elas são capazes de formar biofilmes de difícil remoção em superfícies orgânicas e inorgânicas, e apresentam resistência a diversos métodos químicos de desinfecção (1-3). As micobactérias não tuberculosas podem ser classificadas conforme o tempo de crescimento em meio de cultura, sendo divididas em micobactérias de crescimento lento (mais de 7 dias) e micobactérias de crescimento rápido – menos de 7 dias (4,5).

Infecções por micobactérias de crescimento rápido tem sido reportadas no Brasil, associadas à realização de procedimentos cirúrgicos, notadamente aqueles nos quais se utilizam equipamentos de vídeo (4,6). Entre as micobactérias de crescimento rápido descritas estão as espécies pertencentes ao complexo *M. abscessus*: *M. abscessus* subsp. *abscessus*, *M. abscessus* subsp. *massiliense* e *M. abscessus* subsp. *bolettii*. Os surtos relatados no Brasil descrevem infecções por *M. massiliense*, que até o momento parece ser a subespécie predominante. Das três subespécies conhecidas, o *M. abscessus* é a que apresenta maior perfil de resistência aos antimicrobianos (7).

Em 7 de abril de 2022, a Coordenação de Vigilância Sanitária do Mato Grosso do Sul emitiu alerta técnico sobre surto de infecções por *M. abscessus* em pessoas submetidas a procedimentos estéticos em Pedro Juan Caballero, Paraguai. A situação motivou investigação conjunta conduzida pelos programas de treinamento em epidemiologia de campo do Brasil e Paraguai. Todos os casos estavam vinculados a um único hospital e cirurgião.

Este trabalho tem por objetivo relatar a investigação de campo de surto de infecções cutâneas por *Mycobacterium abscessus* subsp. *abscessus* após procedimentos estéticos.

## Métodos

### Delineamento

Trata-se de estudo de coorte retrospectiva utilizando dados secundários disponibilizados pelo Programa de Epidemiologia Aplicada aos Serviços do SUS (EpiSUS), sobre investigação epidemiológica de campo realizada em 2022, em parceria com o Field Epidemiology Training Program do Paraguai. Os dados constantes no banco foram obtidos durante investigação epidemiológica de campo conduzida em ação de vigilância em saúde pública.

### Contexto

Em abril de 2022, o Ponto Focal Nacional para o Regulamento Sanitário Internacional do Brasil identificou rumor sobre infecções pós-cirúrgicas em pacientes submetidos a cirurgias plásticas em hospital de Pedro Juan Caballero, Paraguai. A informação foi confirmada pela Vigilância Sanitária de Mato Grosso do Sul e pelo Ponto Focal Nacional para o Regulamento Sanitário Internacional do Paraguai, motivando investigação conjunta. A equipe do EpiSUS foi convidada pela equipe do Paraguai para participar das atividades de campo. A investigação foi realizada em Pedro Juan Caballero, departamento de Amambay, cidade que faz fronteira seca com Ponta Porã, estado de Mato Grosso do Sul, região de intenso fluxo transfronteiriço e relevância epidemiológica.

Amostras biológicas obtidas das lesões cutâneas foram encaminhadas ao Laboratório Central de Saúde Pública de Mato Grosso do Sul (Lacen), onde foram processadas por meio de cultivo em meio sólido (Lowenstein-Jensen) e em meio líquido (caldo MGIT). A identificação molecular foi realizada por hibridação com sonda em linha (*line probe assay* – LPA). Posteriormente, as amostras foram encaminhadas ao laboratório de referência da Fundação Oswaldo Cruz (Fiocruz) para confirmação e avaliação de clonalidade. Amostras de água coletadas no estabelecimento foram também processadas no Lacen, com análise microbiológica e tentativa de isolamento de micobactérias de crescimento rápido.

### Participantes

Pessoas submetidas a cirurgias plásticas entre 1º agosto de 2021 e 30 abril de 2022 em hospital privado de Pedro Juan Caballero.

### Variáveis, fontes de dados e mensuração

A fonte de dados utilizada neste estudo foi um banco de dados anonimizado, disponibilizado pelo EpiSUS. Os dados foram coletados durante investigação epidemiológica de campo realizada em contexto de emergência em saúde pública, no âmbito das ações regulares de vigilância em saúde.

Durante a investigação, a população foi definida a partir de uma lista de 108 pessoas submetidas a cirurgias estéticas entre agosto de 2021 e abril de 2022, fornecida pelo profissional responsável pelos procedimentos. A lista incluía nome, tipo de cirurgia, data do procedimento e telefone para contato.

Com base nessas informações, as equipes elaboraram um questionário bilíngue para realização de entrevistas, que abrangeu: identificação (nome, data de nascimento, endereço), dados sociodemográficos (sexo, raça/cor da pele, ocupação, escolaridade, renda), histórico de saúde (comorbidades, uso de medicamentos), dados do procedimento (data, horário, duração, tipo), quadro clínico (data de início dos sintomas, sinais e sintomas apresentados) e informações laboratoriais (data da coleta, tipo de amostra, método laboratorial e microrganismo isolado).

As pessoas foram contatadas por telefone e convidadas a participar da entrevista, conduzida conjuntamente por investigadores do Paraguai e do EpiSUS. Cidadãos paraguaios foram contatados pela equipe do Paraguai e cidadãos brasileiros, pelo EpiSUS. Diversas tentativas de contato foram realizadas em dias e horários distintos, sem limite preestabelecido. Como estratégia de sensibilização, foi enviado previamente um contato via aplicativo de mensagens, com apresentação da equipe investigadora, objetivos do estudo e agendamento da entrevista.

Cada investigador realizou, no mínimo, três tentativas de contato com cada pessoa, por telefone ou aplicativo. Foram consideradas como perdas as situações em que não houve sucesso nas tentativas de contato. Além das entrevistas, foram utilizados dados secundários provenientes da inspeção sanitária realizada pela autoridade sanitária do Paraguai.

No estudo de coorte, foram incluídas 75 pessoas (10 casos confirmados e 65 assintomáticas). Foram testadas possíveis associações entre adoecimento e variáveis socioeconômicas/demográficas e de exposição. O banco de dados utilizado no estudo pode ser acessado no repositório SciELO Data (https://data.scielo.org/).

### Análise estatística

As variáveis numéricas foram descritas por medidas de tendência central e dispersão. As variáveis categóricas foram apresentadas em frequências absolutas. A medida de associação utilizada foi a razão de risco (risco relativo – RR), e as associações foram avaliadas quanto à significância estatística.

As associações entre variáveis categóricas foram analisadas pelo teste exato de Fisher. As análises estatísticas foram realizadas no software Epi Info versão 7.2.5.0, e os gráficos foram elaborados no Microsoft Excel 2016.

## Resultados

Entre agosto de 2021 e abril de 2022, foram realizadas 108 cirurgias por um mesmo cirurgião em hospital privado de Pedro Juan Caballero. Quatorze pessoas (12,9%) apresentaram sintomas compatíveis com infecção por micobactéria de crescimento rápido (abcesso, fístula, deiscência de sutura). Entre elas, 10 (9,3%) tiveram confirmação laboratorial para *Mycobacterium abscessus* subsp. *abscessus* e todas foram entrevistadas. As quatro pessoas sintomáticas restantes não realizaram coleta de amostra para diagnóstico laboratorial. Entre as 94 pessoas assintomáticas, 65 (69,1%) foram localizadas e entrevistadas (Figura 1).

Em dezembro de 2021, foram realizadas 21 cirurgias (19,4%), com quatro casos confirmados (incidência de 0,19). Em janeiro de 2022, foram realizadas 11 cirurgias (10,2%), das quais cinco evoluíram para casos confirmados (incidência de 0,45) (Figura 2).

Todos os casos confirmados (n=10) ocorreram em mulheres, com idades entre 30 e 46 anos (Mediana=34; Q1=33; Q3=42), sem comorbidades relatadas. Nove eram brasileiras e uma, paraguaia. A maioria possuía ensino superior (n=5) ou pós-graduação (n=1), e a renda informada variou entre até 2 salários mínimos e mais de 5 até 10 salários mínimos (quatro não informaram). Quanto à raça/cor da pele, oito se autodeclararam brancas e duas, pardas (Tabela 1).

O início dos sintomas ocorreu entre 3 e 72 dias após a realização do procedimento cirúrgico, com mediana de 31 dias (Q1=21; Q3=65). O primeiro caso teve início em 5 de janeiro de 2022, 36 dias após a realização de lipoabdominoplastia e colocação de próteses mamárias. O último caso foi identificado em 6 de abril de 2022, com antecedente de lipoabdominoplastia realizada em 31 de janeiro (65 dias de incubação).

As manifestações clínicas incluíram formação de nódulos subcutâneos hiperemiados, evoluindo para coloração violácea, fístulas e drenagem purulenta, compatíveis com reação granulomatosa. Oito das 10 pacientes fizeram uso empírico de antimicrobianos antes da confirmação laboratorial. Os antimicrobianos utilizados, os sinais e sintomas clínicos, a idade e o período de incubação dos casos estão descritos na (Tabela 2).

O perfil de sensibilidade das cepas isoladas demonstrou resistência a claritromicina, doxiciclina, sulfametoxazol-trimetoprim, linezolida, ciprofloxacino e moxifloxacino, além de resistência intermediária ao imipenem e à cefoxitina. As cepas foram sensíveis apenas à amicacina, tigeciclina e bedaquilina.

Com base no antibiograma, foi instituído tratamento com bedaquilina (100 mg: 4 comprimidos/dia por 14 dias, seguido de 2 comprimidos/dia três vezes por semana por 24 semanas, via oral), amicacina (500 mg: 2 ampolas/dia por 14 dias, seguida de 2 ampolas/dia três vezes por semana por 8 semanas, via intravenosa) e tigeciclina (50 mg: 2 ampolas no primeiro dia, seguida de 1 ampola/dia por 41 dias, via intravenosa). O tratamento foi realizado no Hospital Universitário de Dourados, Mato Grosso do Sul, para as pacientes brasileiras e no hospital local de Pedro Juan Caballero para a paciente paraguaia.

O risco relativo de adoecer foi maior para aquelas que fizeram cirurgia no período crítico (dezembro a janeiro [RR 6,50; IC95% 2,3; 18,5; p-valor 0,003]) e para aquelas que fizeram lipoabdominoplastia (RR 10,29; IC95% 1,4; 77,2, p-valor 0,004) (Tabela 3).

A Fiocruz realizou avaliação de clonalidade por sequenciamento de genoma total (*whole genome sequencing*) em amostras obtidas dos casos. O laudo revelou alto índice de similaridade genômica entre os isolados, com identificação dos mesmos SNPs (*Single Nucleotide Polymorphisms*, polimorfismos de nucleotídeo único em português), deleções e inserções, indicando que os casos estavam relacionados a uma mesma cepa clonal de *Mycobacterium abscessus* subsp. *abscessus.*

Resultados de amostras ambientais apontaram que a água utilizada no estabelecimento não recebia tratamento e, portanto, não era clorada. Foram encontrados coliformes totais em dois pontos (saída do reservatório e uma das torneiras do lavatório do centro cirúrgico) e *Pseudomonas aeruginosa* em outros três pontos (torneira do expurgo e duas torneiras do lavatório do centro cirúrgico). Alíquotas da amostra foram semeadas para isolamento de micobactérias de crescimento rápido, com resultado negativo.

O estabelecimento foi inspecionado pela autoridade sanitária do Departamento de Amambay, Paraguai, com apontamento de inconformidades e sugestões de melhoria. Entre outras informações, o relatório produzido pela equipe de inspeção paraguaia relata: fragilidades nos protocolos de higiene; manejo incorreto de resíduos infectantes; desorganização e falta de limpeza na sala de esterilização; falta de manutenção em aparelhos de ar-condicionado; falta de sala de isolamento; existência de arranjos de flores artificiais; e identificação e rotulagem imprecisas de materiais esterilizados.

Em uma das visitas, houve relato verbal de que a profissional responsável pela limpeza dos materiais foi afastada por questões de saúde em meados de dezembro de 2021, sendo substituída por outra trabalhadora.

## Discussão

Este estudo descreve um surto de infecção por *Mycobacterium abscessus* subsp. *abscessus* após cirurgias estéticas realizadas em hospital privado de Pedro Juan Caballero, Paraguai. Houve associação entre a ocorrência de infecção e a realização de cirurgias nos meses de dezembro de 2021 e janeiro de 2022, e realização de lipoabdominoplastia. A análise genômica identificou se tratar de uma mesma cepa entre os casos, reforçando a hipótese de uma fonte comum de infecção.

Os resultados devem ser considerados à luz das limitações inerentes ao uso de dados secundários e ao delineamento retrospectivo, que pode estar sujeito a vieses de seleção e de memória. A definição da população exposta se baseou em registros fornecidos pelo próprio profissional responsável pelas cirurgias, o que pode ter levado à subnotificação de procedimentos realizados. A possibilidade de viés de informação também deve ser considerada, já que os dados foram coletados por entrevistas retrospectivas, conduzidas em contextos distintos e com potenciais diferenças de compreensão entre os entrevistados.

A associação entre o período crítico (dezembro-janeiro) e o aumento de casos sugere uma falha pontual nos processos de esterilização e controle de infecção. A substituição da profissional responsável pela limpeza dos materiais em dezembro, relatada durante a inspeção sanitária, pode ter representado um momento de vulnerabilidade. A adequada limpeza de materiais reutilizáveis, como cânulas de lipoaspiração, exige conhecimento técnico e protocolo rigoroso de reprocessamento (8), o que pode não ter sido plenamente assegurado no contexto identificado. Estudos brasileiros anteriores já descreveram surtos de micobactérias de crescimento rápido relacionados a falhas no reprocessamento de materiais, com destaque para a resistência dessas micobactérias à remoção mecânica e a saneantes comumente utilizados, como o glutaraldeído (9-12).

O padrão clínico observado, com sintomas de início tardio e evolução para lesões fistulizadas, é compatível com o comportamento de infecções por micobactérias de crescimento rápido após procedimentos invasivos. A resistência antimicrobiana encontrada nos isolados reforça os desafios terapêuticos associados a esses agentes, como já descrito em surtos ocorridos no Brasil entre 1998 e 2017, em que o tratamento foi prolongado, de alto custo e, muitas vezes, com necessidade de reabordagem cirúrgica (13). A sensibilidade restrita a três antimicrobianos – tigeciclina, bedaquilina e amicacina – limitou as opções terapêuticas disponíveis, exigindo articulação interinstitucional para viabilizar o tratamento, especialmente no caso de pacientes brasileiras.

A investigação genômica indicou que os isolados clínicos pertenciam a uma mesma cepa clonal, o que corrobora a hipótese de fonte comum. O achado é compatível com estudos conduzidos em outros cenários, que também identificaram clonalidade entre isolados clínicos em surtos relacionados a procedimentos estéticos (11,14,15). Embora a coleta de amostras ambientais tenha sido negativa para micobactérias de crescimento rápido, a identificação de coliformes e *Pseudomonas aeruginosa* em pontos críticos da unidade hospitalar sugere falhas sistêmicas no controle sanitário da água, o que pode ter contribuído para o contexto de contaminação. A ausência de cloração na água utilizada no hospital reforça essa interpretação.

O crescimento do turismo médico, impulsionado por fatores como custos reduzidos, menor tempo de espera e busca por discrição, tem sido acompanhado por aumento no número de eventos adversos (16,17). Relatos internacionais de infecção por micobactérias de crescimento rápido após cirurgias plásticas realizadas em países como República Dominicana e México mostram similaridades com o esse surto, incluindo o perfil demográfico dos casos, o tipo de procedimento e a resistência antimicrobiana dos isolados (18-20). Embora o Brasil já tenha registrado casos de infecção por micobactérias de crescimento rápido após procedimentos estéticos, não foram encontrados relatos anteriores envolvendo brasileiros expostos fora do território nacional. O surto atual configura um alerta para as autoridades sanitárias de ambos os países.

O surto investigado apresenta elementos característicos de infecção relacionada à assistência à saúde, incluindo fator de risco individual (tipo de cirurgia), fator temporal (concentração dos casos em curto intervalo) e fator ambiental (infraestrutura e práticas sanitárias inadequadas). É possível que os casos tenham apresentado infecção polimicrobiana inicialmente, e que o uso empírico de antimicrobianos antes da confirmação diagnóstica possa ter contribuído para a seleção de *M. abscessus*, cuja resistência a múltiplas classes terapêuticas é documentada (2,7).

Conclui-se que esse surto esteve associado a falhas sanitárias em estabelecimento de saúde privado, agravadas por contexto de turismo médico. Os achados reforçam a necessidade de mecanismos eficazes de regulação, vigilância e cooperação internacional para a prevenção de eventos adversos em procedimentos transfronteiriços.

**Figura 1.** População de estudo, entrevistas realizadas e situação clínica segundo presença de sintomas. Paraguai, 2021-2022 (n=108)

**Figura 2.** Distribuição de cirurgias estéticas, casos confirmados e início dos sintomas segundo semana epidemiológica. Paraguai, 2021-2022 (n=108)

**Tabela 1.** Características sociodemográficas de casos confirmados e pessoas assintomáticas. Paraguai, 2021-2022 (n=75)

Variável	Confirmados (n=10)	Assintomáticos (n=65)	Total
	n	n	(n=75)
**Sexo**			
Feminino	10	61	71
Masculino	-	4	4
**Faixa etária (anos)**			
Menor de 30	-	19	19
30-39	6	24	30
40-49	4	14	18
50 e mais	-	7	7
Ignorado	-	1	1
**Raça/cor da pele**			
Branca	8	37	45
Preta	-	2	2
Amarela	-	1	1
Parda	2	22	24
Não sabe	-	3	3
**Escolaridade**			
Fundamental	2	2	4
Médio	2	21	23
Superior	5	37	42
Pós-graduação	1	5	6
**Nacionalidade**			
Brasileira	9	50	59
Paraguaia	1	15	16
**Renda**			
Até 2 salários	3	11	14
Mais de 2 até 5	2	24	26
Mais de 5 até 10	1	5	6
Mais de 10	-	3	3
Não informado	4	22	26

**Tabela 2.** Casos confirmados de acordo com idade, período de incubação, sinais e sintomas apresentados e antimicrobiano utilizado. Paraguai, 2021-2022 (n=10)

Característica	Caso									
	1	2	3	4	5	6	7	8	9	10
**Idade (anos)**	33	34	32	41	30	36	31	42	46	43
Período de incubação (dias)	36	21	30	3	20	70	36	72	65	21
**Sinais e sintomas**	Nódulo doloroso, hiperemia, aumento de calor local, fístula, secreção	Nódulo indolor, hiperemia, aumento de calor local, fístula, secreção	Nódulo doloroso, hiperemia, aumento de calor local, fístula, secreção	Nódulo doloroso, hiperemia, aumento de calor local, secreção, deiscência	Nódulo doloroso, hiperemia, calor local, fístula, secreção, deiscência	Nódulo doloroso, hiperemia, calor local, fístula, secreção, deiscência	Dor local, hiperemia, calor local, fístula, secreção, deiscência	Nódulo (inicialmente indolor, evoluindo com dor local)	Fístula, secreção	Secreção, deiscência
**Antibióticos utilizados**	1. claritromicina 500mg 12/12h por 15 dias 2. cefuroxima 500mg 12/12h por 15 dias 3. ciprofloxacino 500mg 12/12h por 75 dias 4. claritromicina 500mg 8/8h por 75 dias 3. fluconazol	1. azitromicina 500mg 1x/dia por 6 dias	1. ciprofloxacino 500mg 12/12 por 20 dias 2. claritromicina 500mg 8/8h por 20 dias 3. fluconazol 150mg 1 cápsula/semana por 4 semanas	1. amoxicilina/clavulanato	1. ciprofloxacino 500mg 12/12h por 30 dias 2. claritromicina 500mg 8/8h por 30 dias	1. ciprofloxacino 500mg 12/12h por 20 dias 2. claritromicina 500mg 12/12h por 20 dias 3. ciprofloxacino 500mg 12/12h por 30 dias 4. doxiciclina 200mg 12/12h por 30 dias	1. sulfametoxazol/trimetoprim 12/12h por 15 dias 2. claritromicina 500mg 8/8h por 15 dias 3. ciprofloxacino 500mg 12/12h por 15 dias 4. axetilcefuroxima 12/12h por 7 dias 5. metronidazol 400mg 8/8h por 7 dias	1. claritromicina 500mg 8/8h por 14 dias	Não fez uso de ATB	Sem informação

**Tabela 3.** Associação entre características sociodemográficas, tipo de cirurgia e ocorrência de casos confirmados, com risco relativo (RR [IC95%]). Paraguai, 2021-2022 (n=75)

Variável	Confirmados	Assintomáticos	RR (IC95%)	p-valor
**Sexo**				1,000
Feminino	10	61	0,90 (0,14; 5,87)	
Masculino	0	4		
**Renda**			** **	** ** 0,334
Até 2 salários	3	11	1,95 (0,76; 5,05)	
Mais de 2 salários	3	32		
**Escolaridade**			** **	** ** 1,000
Fundamental/médio	2	15	0,85 (0,19; 3,64)	
Superior	8	50		
**Raça/cor da pele**				0,302
Branca	8	37	2,40 (0,54; 10,48)	
Não branca	2	25		
**Período crítico**	** **			0,003
Sim	5	5	6,50 (2,28; 18,49)	
Não	5	60		
**Mais de 1 procedimento**				1,000
Sim	2	12	1,09 (0,26; 4,58)	
Não	8	53		
**Tipo de cirurgia**			** **	** **
**Lipoabdominoplastia**				0,004
Sim	9	26	10,29 (1,37; 77,19)	
Não	1	39		
**Mastopexia**				1,000
Sim	1	8	0,81 (0,12; 5,69)	
Não	9	57		
**Prótese mamária**				0,480
Sim	2	23	0,50 (0,11; 2,18)	
Não	8	42		

## Data Availability

O banco de dados utilizado no estudo pode ser acessado no repositório SciELO Data (https://data.scielo.org/).
